# A primer on using balancing weights to estimate inverse probability weights

**DOI:** 10.1093/aje/kwaf060

**Published:** 2025-04-07

**Authors:** Haedi E Thelen, Todd A Miano, Luke Keele

**Affiliations:** Department of Biostatistics, Epidemiology and Statistics, University of Pennsylvania, Philadelphia, PA, United States; Department of Biostatistics, Epidemiology and Statistics, University of Pennsylvania, Philadelphia, PA, United States; Department of Pharmacy, Hospital of the University of Pennsylvania, Philadelphia, PA, United States; Center for Surgery and Health Economics, Perelman School of Medicine, Philadelphia, PA, United States

## Weighting methods

A key challenge in observational studies aiming to estimate causal effects is avoiding bias from confounding (ie, differences in baseline risk for the outcome between groups being compared). Control of confounding is often achieved through statistical adjustment, which can render treated and comparator groups comparable in terms of baseline covariates. Regression models were long the primary method of statistical adjustment. Many alternatives are now available, including the parametric g-formula and a variety of matching methods and weighting methods. Weighting methods have become perhaps the most popular approach in epidemiology.

Weighting methods are most often implemented using logistic regression to estimate inverse probability of treatment weights (IPTWs), as described by several authors.^1-3^ Hereafter, we refer to these as model-based weights. Model-based weights, however, 1) may fail to balance covariate distributions without iterative modifications to the model, especially as more covariates are included; and 2) can produce extreme weights, which leads to uncertainty and bias in effect estimates.^1^^,^^4^ This brief methods review describes balancing weights as an alternative method for estimating IPTWs that can overcome the limitations of model-based weights. We illustrate their use in an application. We focus on the setting where we aim to estimate the average treatment effect of a binary point exposure on an outcome. Throughout, we assume the usual assumptions of conditional exchangeability, consistency, and positivity hold.^5^

### Model-based weights

Inverse probability of treatment weights are calculated using an individual’s probability of receiving treatment, often referred to as the propensity score. Model-based estimates of the propensity score are based on a logistic regression model that regresses treatment status on the baseline covariates. Treated patients are then weighted by the inverse of the probability of being treated, and comparator patients are weighted by the inverse probability of not being treated. The likelihood function for logistic regression is designed to maximize the probability of the observed data. In this case, logistic regression seeks to predict treatment status based on covariate values. It is not designed to minimize covariate imbalance. Once estimated, the investigator performs diagnostic checks to examine overlap of the estimated propensity scores (as a measure of positivity) and to determine whether the resulting IPTWs adequately balance covariates. These diagnostics inform whether model revision or weight truncation is needed before estimating treatment effects.

Model-based weights may perform poorly in a number of ways. First, imbalances may remain in covariate distributions because model-based weights indirectly target covariate balance. Second, model-based estimation may produce extremely large weights. Logistic regression is sensitive to outliers, and outliers, which are found in areas of weak overlap, often cause weights to become very large or very small, leading to larger bias in final effect estimates. Extreme weights can be truncated or trimmed and overlap weights can be used.^4^^,^^6^ However, these approaches change the causal estimand and may limit the generalizability of results.^1^

### Balancing weights

Balancing weights are an alternative estimation method for IPTWs; the former are designed to directly target balance among covariates. There are many approaches for estimating balancing weights. However, they are all based on an optimization problem that minimizes imbalance subject to a constraint on the dispersion of the weights.^1^^,^^3^^,^^7^ At a high level, balancing weights are the set of weights that minimize the following objective function (adapted from Ben-Michael et al. 2021)^2^:


(1) \begin{equation*} \mathit{\min}\left[{imbalance}_M+ hyperparameter\times weight\ dispersion\right] \end{equation*}

where ${imbalance}_M$ represents how imbalance is modeled. Various implementations of balancing weights use different metrics to summarize imbalance (eg, sum of squared difference in means or measuring the maximum imbalance across all covariates) and weight dispersion (eg, the sum of squared weights or entropy).^2^ The hyperparameter governs the trade-off between minimizing imbalance and dispersion.^2^ A small hyperparameter value will prioritize minimizing imbalance, allowing more dispersed weights, whereas a larger value will allow for some residual imbalance but reduce dispersion. In short, the hyperparameter controls the bias-variance trade-off in treatment effect estimation. In general, hyperparameter selection is accomplished by evaluating a range of values and choosing 1 that gives acceptable balance and dispersion.^8^ Other data-driven methods have been proposed. For example, in the illustration we present next, we set the hyperparameter to the variance of residuals in a regression of the outcome on the covariates in the comparator group.^7^

As with model-based weights, investigators must specify the model form (${imbalance}_M$). That is, they must specify whether interactions or higher-order terms must also be balanced. This can be done using knowledge of covariate relationships or machine-learning methods.^7^ Once balancing weights are estimated, the analysis proceeds as for model-based weights. The investigator checks for extreme weights and balance in the weighted population. If satisfied, the analysis proceeds. Balancing weights can be used in place of model-based IPTWs in marginal structural models and in augmented-IPTW estimators, enjoying all the usual doubly robust properties.^2^ An immediate benefit of using balancing weights is that they tend to outperform model-based IPTWs in both simulation and application studies, particularly in settings with weak overlap.^1^^,^^6^^,^^7^

## Illustration

### Methods

**Figure 1:**
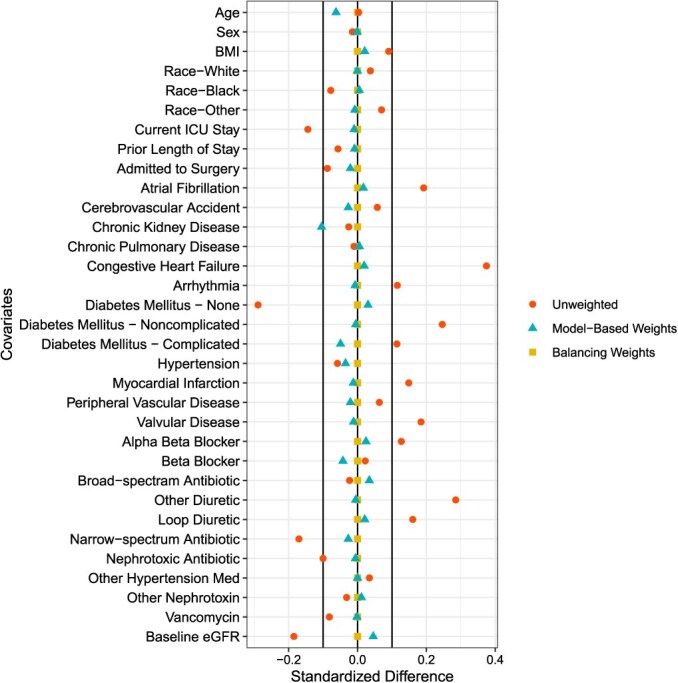
Balancing weights vs model-based weights. Standardized mean differences are shown for the unweighted data, data weighted with model-based weights, and with the balancing weights when the hyperparameter was set to 0.07. Balancing weights achieve near-perfect balance for all covariates, whereas weights selected through model-based methods leave residual imbalance for the majority of covariates. Abbreviations: BMI, body mass index; eGFR, estimated glomerular filtration rate; ICU, intensive care unit; Med, medication.

Here, we illustrate the use of balancing weights to determine the risk of acute kidney injury in hospitalized patients treated with renin-angiotensin system inhibitors (the exposure of interest) compared with those treated with calcium channel blockers (the active comparator). We compare balancing weights with model-based weights. This example uses data from a retrospective cohort study of hospitalized patients treated with nonsteroidal anti-inflammatory drugs, as described by Miano et al.^9^ Briefly, patients were followed from initiation of medication for the outcome of in-hospital acute kidney injury. The 33 covariates listed in Figure 1 were measured before starting follow-up.

For both approaches, we assume all relationships among covariates and treatment assignment are linear—no splines, interactions, or higher-order terms were included. Model-based weights were estimated using logistic regression fit only once (ie, we did not iterate over specifications of this model). Balance weights were estimated using the method implemented in the R library (namely, the *balancer* package), whereby imbalance is summarized using the sum of squared differences in means and weight dispersion is measured as the sum of squared weights.^6^^,^^7^ We set the hyperparameter using the data-driven approached described above.

To compare performance of the methods, we measured balance using standardized mean differences (SMDs). The SMDs of covariates between treated and comparator groups before and after weighting were calculated, where an absolute SMD < 0.1 was considered imbalanced. Percent bias reduction (PBR) was used to summarize performance across all the SMDs. The PBR was calculated as a summary measure of how much imbalance is reduced for the full set of *K* covariates:


(2) \begin{equation*} PBR=\left[{}^{\frac{1}{k}{\sum}_k\mid{SMD}_{Weighted}\mid }\!\left/ \!{}_{\frac{1}{k}{\sum}_k\mid{SMD}_{Unweighted}\mid}\right.\right]\times 100\% \end{equation*}

Dispersion of weights was measured using effective sample size (ESS). For a sample with weights {*w_1_, … w_n_*}, $ESS={\left({\Sigma}_{i=1}^n\ {w}_i\right)}^2/\left({\Sigma}_{i=1}^n\ {w}_i^2\right)$, where a larger ESS, closer to the unweighted data set’s sample size, is preferred.^1^ A low ESS often indicates several highly influential observations with extreme weights.^1^ Code and data to repeat all analyses are available on GitHub (https://github.com/HaediThelen/BalWts).

## Results

The majority of covariates were imbalanced in the unweighted data, with the some of the greatest imbalances (>0.35) indicating areas of weak overlap and potential violations of positivity. The PBR for model-based weights was 81%. Balancing weights achieved excellent balance for all covariates, resulting in a PBR > 99% (Figure 1 and Table 1). In this illustration, balancing weights left less residual imbalance between groups than did model-based weights, and they had a larger ESS. Thus, in this case, using balancing weights to estimate IPTWs provides greater control of confounding than model-based weighting methods. The larger ESS with the balance weighting method provides additional precision in the final effect estimates.

**Table 1:** Comparing methods to estimate weights in a population of hospitalized patients exposed to study drugs, Philadelphia, PA, 2004-2017.

	**Original sample**	**Model-based weights**	**Balancing weights** ^a^
PBR, %	–	81	99.99
ESS, no.	5431	4992	5020

Abbreviations: ESS, effective sample size; PBR, percent bias reduction.

a Balancing weights have larger PBR model-based weights, reducing bias in the final estimates. The balancing weights yield a larger total ESS after weighting than do model-based weights, which implies less variance in the weight and, ultimately, more precision in final outcome estimates. The hyperparameter used to estimate balancing weights, 0.07, was selected for this analysis through the data-driven approach.

## Discussion

Balancing weights are an alternative to model-based IPTWs. They can be used in place of model-based weights in any setting; however, balancing weights are particularly useful in settings where model-based weights have difficulty balancing covariates without becoming extreme. In particular, they are useful in data sets with weak overlap. Overlap weights are increasingly used in this setting, however, because they limit the causal estimand to those within the overlap region—the generalizability of results can be limited. Balancing weights have been shown to balance covariates in limited overlap settings where model-based weights could not.^6^ In these instances, overlap weights can be avoided and the interpretation of results does not need to change.^6^

One limitation of balancing weights is that, like model-based weights, they require the investigator to specify the model form, and errors in this step can lead to model misspecification and bias. Doubly robust machine-learning estimators are an alternative method and have the advantage that the model form does not need to be specified.^10^

Balancing weights can achieve excellent covariate balance, even in settings of weak overlap, while avoiding extreme weights that increase variance in effect estimates. They do this in a single step—no iteration and model respecification are required. They are an attractive option for investigators using weighting methods to estimate treatment effects in epidemiologic studies.

### Further readings

For more in-depth summaries, we refer readers to Chattopadhyay et al 2020,^1^ Zubizarreta et al. 2023,^3^ and Ben-Michael et al., 2021.

## Data Availability

Data and R code to repeat analyses are publicly available at https://github.com/HaediThelen/BalWts.
